# Increased Neural Activity in Hazardous Drinkers During High Workload in a Visual Working Memory Task: A Preliminary Assessment Through Event-Related Potentials

**DOI:** 10.3389/fpsyt.2019.00248

**Published:** 2019-04-18

**Authors:** Elisa Schroder, Clémence Dousset, Xavier Noel, Charles Kornreich, Salvatore Campanella

**Affiliations:** Laboratoire de Psychologie Médicale et d’Addictologie, ULB Neuroscience Institute (UNI), CHU Brugmann-Université Libre de Bruxelles (U.L.B.), Brussels, Belgium

**Keywords:** heavy social drinking, alcohol, working memory, cognitive workload, n-back task, event-related potentials

## Abstract

Despite equated behavioral performance levels, hazardous drinkers generally exhibited increased neural activity while performing simple cognitive tasks compared to light drinkers. Here, 49 participants (25 hazardous and 24 light drinkers) participated in an event-related potentials (ERPs) study while performing an *n*-back working memory task. In the control zero-back (N0) condition, the subjects were required to press a button when the number “2” or “6” was displayed. In the two-back and three-back (N2; N3) conditions, the subjects had to press a button when the displayed number was identical to the number shown two/three trials earlier. To assess for the impact of alcohol consumption on the updating of working memory processes under various cognitive loads, difference waveforms of “N2 minus N0” and “N3 minus N0” were computed by subtracting waveforms in the N0 condition from waveforms in the N2 and N3 conditions, for the light and the hazardous drinkers. Three main ERP components were noted for both groups: a P200/N200 complex, a P300 component, and an N400/P600 activity. The results show that, to perform the task at the same level as the light drinkers, the hazardous drinkers exhibited larger amplitude differences, mainly around the P300 and P600 components. These data may be considered, at the preventive level, as vulnerability factors for developing adult substance use disorders, and they stress the importance, at a clinical level, to consider such working memory processes in the management of alcohol dependence.

## Introduction

Working memory (WM), the capacity to store information in short-term registers and simultaneously manipulate it online, is required for most key daily living activities such as planning, engaging in active conversation, or solving complex problems (1). At the functional level, three categories covering most of the functions indexing WM have been well-described: storage and processing, executive processes, and coordination (2). Indeed, one of the main characteristics of WM refers to a capacity limited mental workspace used to store and process information for use in ongoing cognition (3). This WM load is, therefore, a reasonable measure of the cognitive effort dedicated to holding information in mind for short periods of time while performing a cognitive task (4). It was traditionally proposed by Miller (5) as the “magical number seven plus or minus two” items that can be remembered. Neuroimaging studies have shown that when the WM load exceeds the individual short-term memory capacity, the dorsal prefrontal cortex (PFC)—in addition to ventral PFC regions—may be recruited to mediate strategic processes necessary for the maintenance of a high WM load [e.g., Ref. (6)]. In recent years, there has been considerable debate regarding the notion of a capacity limitation in WM as well as on whether mechanisms of interference, rather than capacity limits, might explain performance limitations [e.g., see Ref. (7)]. Also, WM executive processes refer to three main functions identified as mental set shifting (e.g., the ability to shift from one task to another one), inhibition of prepotent responses (e.g., the ability to suppress a dominant motor response), and information updating (e.g., the ability to update relevant information compared to nonrelevant ones in WM) (8). A third important role of WM is to coordinate elements and build new relations to integrate them into structures (e.g., representing different visual objects in a three-dimensional space). These three different functional facets can be isolated and described on their own. Nevertheless, WM functions as a whole, and all these different facets interact for higher level processes (2). The main point we will focus on here is that *individual differences in WM load* correspond to fundamental differences in *executive control skills* [e.g., Ref. (9)] that might impact some dysfunctional behaviors such as impulsive decision-making typically observed in addictive behaviors [e.g., Ref. (10)].

In dual-process neurocognitive models, the persistence of heavy alcohol consumption results from a) an abnormal bottom-up system generating craving and automatic alcohol-approach tendencies; paired with b) an abnormal top-down system generating reduced cognitive control upon long-term prospects [e.g., Ref. (11)]. The underlying neural mechanisms of these phenomena are defined by increased dopamine release in the cortico‐striatal reward circuit triggered by drug stimuli [e.g., Ref. (12)], which draws the subject’s attention to the drug-related stimulus [e.g., Ref. (13)], while hypoactivation of frontal regions indicates that alcoholics lack the executive resources needed to inhibit the salient and dominant response [e.g., Ref. (14)]. In this view, a lot of empirical research has been devoted to the role of neurocognitive processes such as cue reactivity [e.g., see Ref. (15) for a meta-analysis] or inhibitory skills [e.g., see Ref. (16) for a review] in the onset, development, and persistence of heavy alcohol consumption. Indeed, both of these processes appear to be promising targets for interventions aimed at treating patients with alcohol disorder [e.g., Refs. (17, 18)].

However, WM capacity has also been shown to impact cognitive control of impulsivity by way of keeping future goals in mind when making decisions when faced with rewarding/arousing distractions (19). This fits perfectly with the dual-process model of cognitive control, whereby executive functions are used to regulate bottom-up implicit arousal responses (14, 20). Indeed, a threshold of PFC activation is needed for effective modulation of bottom-up processes, and is associated with WM [e.g., Ref. (21)]. In such a view, low WM capacity can exacerbate the worse impulse control that results from excessive consumption of alcohol [e.g., Ref. (22)], by triggering poor inhibition of immediate behavior as well as poor longer-term planning of future options (10). Chronic heavy users of alcohol often exhibit lower levels of WM capacity [e.g., Refs. (23, 24)]. However, although some of these deficits appear to result from heavy alcohol use [e.g., Ref. (25)], there is also evidence suggesting that low capacity WM problems contribute to the development of alcohol abuse [e.g., Ref. (26)]. WM deficits are then considered to contribute to the core pathology of addiction [e.g., Ref. (14)]. Indeed, Brooks and colleagues conducted a review yielding 93 studies that examined WM and cognitive control, between 2010 and 2017, in patients with substance use disorders (SUD; including stimulants such as nicotine, opioids, and marijuana, and alcohol use). The majority of the studies (72%) reported worse WM performances compared to healthy drug-naive controls or nondrug-taking control groups. From these insights, training WM has been shown to be highly relevant for reducing stimulant (27) as well as alcohol use [e.g., Ref. (28)] by increasing control over automatic impulses, even though different training techniques appear to produce differential impacts on the broader landscape of cognitive abilities (3). Indeed, there is some evidence that suggests that nonsequential and nonadaptive training paradigms should not be effective (29), while “core training programs” using tasks that commonly involve sequential processing and frequent memory updating appear to produce more far-reaching transfer effects, most likely because they target domain-general mechanisms of WM (3). A good illustration of such a training program relates to *the n-back task*, which requires continuous upgrades of the memory store (i.e., a memory updating process) and which is particularly suited for the study of varying levels of WM load (30).

This *n*-back task requires online monitoring, updating, and manipulation of remembered information, and it is, therefore, assumed to place great demands on a number of key processes within WM subtended by widespread neural areas (31). Indeed, frontal regions have been implicated in numerous cognitive functions that are relevant to the *n*-back task, including monitoring and manipulation within WM (32); the parietal cortex is thought to be involved in the implementation of stimulus-response mapping (33) and in the storage of WM contents (34) as a kind of “buffer for perceptual attributes” (35); while activation of the precuneus during the visual WM task is consistent with a recollection process aided by visual imagery (36), and insula activation is considered to be a part of the inferior frontoparietal network, which responds to behaviorally relevant rather than to expected stimuli (37).

This task has been extensively tested in heavy alcohol users to outline WM disturbances linked with high workloads, but it yielded heterogeneous results. Indeed, decreased PFC activation and worse WM were observed, for instance, in adolescent alcohol users [e.g., Ref. (38)] and in youths with a family history of alcoholism (FHA) (39). However, while many functional magnetic resonance imaging (fMRI) studies have reported insignificant differences in behavioral performances between healthy control groups and heavy alcohol users, significant neural differences can be discerned by including brain imaging measures [e.g., Refs. (40–42)], indexing compensatory neural processing during variation in cognitive load (43). The bulk of the reported data consisted of *reduced* activation of the PFC network (including insula, cerebellar, anterior cingulate, and/or parietal regions) in alcoholic patients [e.g., Refs. (40, 44)] or (conversely) *increased* PFC network activation in heavy social drinkers (i.e., people characterized by excessive alcohol consumption, without a clinical state of dependence) (42, 45, 46). According to a “functional compensation view,” decreases or absences in activation reflect deficits in brain function, and the concomitant increases in activation reflect “attempted” or “successful” compensation for these deficits (47). Aside from fMRI studies, differences in electrophysiological [electroencephalogram (EEG)] components are considered to be sensitive indicators of workload (48, 49). Indeed, a decrease in alpha power is associated with an increase in arousal, resource allocation, or workload [e.g., Ref. (50)], and an increase in theta power (most profound over frontal electrodes) has been observed as task requirements increase [e.g., Ref. (51)]. Event-related potentials (ERPs), derived from EEGs, also convey relevant information about an individual’s workload. Throughout the information processing stream, ERP components such as the P100 [e.g., Ref. (52)], the N100 [e.g., Ref. (53)], the N200 (54), a positive/negative component between 140 and 280 ms (55), and the P300 [e.g., Ref. (56)] have been shown to be modulated by the WM workload and task difficulty. By using a visual task with a high WM load (57, 44) or through a spatial 2-back task (58), several ERP studies have determined that memory load capacity is affected in heavy users of alcohol.

However, to our knowledge, there has not been a study to date that specifically investigated the impact of *increasing visual memory load* on neural activity in healthy vs. heavy alcohol users based on ERPs. In light of its high temporal resolution, we sought to define whether increasing WM visual load specifically impacts hazardous vs. light drinkers at specific time intervals throughout the information processing stream. To address this, we chose 1) to use a visual WM *n*-back task (N = 0; 2; or 3), forcing subjects to continuously remember the last two or three rapidly changing items, to induce different levels of visual workload; and 2) to compare light versus heavy social drinkers, as done previously in an fMRI experiment (only comparing N2 vs. N0 conditions) suggesting increased pre-supplementary motor area, PFC, and cerebellar activations in heavy drinkers despite similar behavioral performances (42). In the present ERP study, increasing memory load was applied to participants through N2 and N3-back tasks, and this parametric manipulation of the task variable (visual memory workload) was compared in light vs. heavy alcohol drinkers by use of a subtraction method (N2 minus N0; N3 minus N0) that is well-known to index specific WM processes such as storage and manipulation (updating) (34). Light and hazardous drinkers were enrolled in the study as our aim was to show the potential differences induced by different alcohol consumption patterns (rather than between drinkers and nondrinkers). This strategy appears to be congruent with most earlier studies on heavy social drinking (e.g., cited in this paper) (57, 59), where the control group was composed of light drinkers. Moreover, recent studies have shown that control teetotalers appear to represent a specific population that results in unexpected results (e.g., worse executive performance) (60), which constitutes an additional reason to avoid including nondrinkers in the present study. Our main hypotheses are that 1) light and heavy alcohol drinkers will exhibit similar behavioral performances [see Ref. (61) for a review]; and 2) compared to light drinkers, the higher the memory load, the more that heavy drinkers will recruit neural resources. Moreover, as a result of the optimal temporal resolution of ERPs compared to fMRI (62), a precise temporal window can be defined for this enhanced neural activity recruitment. Such results could have the highest relevance at a prevention level, as these under-investigated WM load processes (compared to executive or cue-reactivity ones) in alcohol disorders could index “biological vulnerability factors” that may trigger further onset of alcohol dependence.

## Materials and Methods

### Participants

First, we conducted a general screening of 120 students from the Faculty of Psychology of the University of Brussels (Belgium) in order to ascertain sociodemographic variables (age, gender, education level, and native language) and patterns of alcohol consumption. On the basis of these self-reported data, groups of participants were defined as detailed below. Exclusion criteria for participants included major medical issues, conditions relating to impairment of the central nervous system (including epilepsy and a prior history of brain injury), visual impairments, and past or current drug consumption (other than alcohol and tobacco use). Our main objective was to select two groups of participants who only exhibited differences in terms of their alcohol-drinking patterns (see **Table 1** for the complete descriptive data). Therefore, subjects concurrently consuming cannabis (defined as at least once in the month prior to the study) were not included. Also, a similar number of participants with a family history of alcoholism (FHA) (63) were included in the final groups (only one by group). In line with earlier studies [e.g., Refs. (42, 59, 64, 65)], three variables (self-reported by participants through the use of a timeline follow-back method questionnaire assessing alcohol–drug consumption characteristics) were used to determine control and heavy alcohol user groups: the mean number of drinking occasions per week (DOW: “how many times do you typically consume alcohol in a week?”), the mean number of alcohol doses per drinking occasion (ADO: “how many drinks do you generally consume during one drinking occasion?”), and the mean number of alcohol doses per week (ADW: “how many drinks do you generally consume in a week?”; one dose corresponding to 10 g of pure ethanol). According to the definition of binge drinking used in European countries, participants who drank six or more standard alcoholic drinks (10 g of alcohol) on the same occasion at a rate of at least two drinks per hour and at most two or three times per week were classified as hazardous drinkers. Those who drank 1 to 30 days a month, but never more than five standard alcoholic drinks on the same occasion and at a maximum rate of two drinks per hour, were classified as controls. This classification was confirmed utilizing the AUDIT-C consumption subscore, which is defined by three items of the complete 10-item AUDIT instrument (66), and which can help identify people who are hazardous drinkers (67). The AUDIT-C is scored on a scale 0–12. A score of 3 for women and 4 for men is considered optimal for identifying hazardous drinkers; the higher the score, the more likely the drinking pattern affects the participants’ safety (68). Hazardous drinking, which can significantly impact public health despite the absence of any *bona fide* disorder in the individual users, is defined as a level of alcohol consumption that is likely to result in harm to the user or other individuals (69).

**Table 1:** The light and the hazardous drinkers were equivalent in terms of age, gender, depression [Beck Depression Inventory (BDI)-II scores], anxiety [State-Trait Anxiety Inventory (STAI)-trait and STAI-state scores], and impulsivity [Urgency Premeditation Perseverance and Sensation Seeking Impulsive Behavior Scale (UPPS) total score] (all *p*’s > 0.05). The two groups differed solely on alcohol variables: the Alcohol Use Disorders Identification Test - Alcohol Consumption questions (AUDIT-C) subscore [*t*(47) = −10.836; *p* < 0.001], the mean number of alcohol doses per drinking occasion (ADO), the mean number of drinking occasions per week (DOW), and the mean number of alcohol doses per week (ADW).

	Light drinkers (*n* = 24)	Hazardous drinkers (*n* = 25)	*T* value	*P* value
Age	26.79 ± 9.3	23.96 ± 2.4	1.442	0.161
Gender (M/F)	11/13	13/12	χ² = 0.186	0.666
AUDIT-C	2.92 ± 1.2	6.76 ± 1.2	−10.836	<0.001
ADO	0.95 ± 0.6	2.12 ± 1.3	−3.977	<0.001
DOW	1.71 ± 1.2	5.16 ± 2.4	−6.236	<0.001
ADW	1.8 ± 1.5	7.3 ± 4.9	−5.255	<0.001
BDI-II	6.92 ± 4.9	5.36 ± 4.3	1.171	0.248
STAI-trait	46.54 ± 8.8	44.72 ± 9.1	0.712	0.48
STAI-state	46.67 ± 9.6	43.92 ± 6.4	1.177	0.245
UPPS	101.67 ± 12.1	105.36 ± 11.195	−1.108	0.273

In order to ensure that any potential difference in the ERP data would be due to alcohol consumption and not to other variables, the groups were balanced for age, gender, and level of education (i.e., the number of years of education completed since starting primary school). The participants were also asked to fill out questionnaires assessing psychological measures. These were the State-Trait Anxiety Inventory (STAI A and B) to assess state and trait anxiety (70); the Beck Depression Inventory (BDI-II) (71) to assess depression; and the Urgency Premeditation Perseverance and Sensation Seeking Impulsive Behavior Scale (UPPS) (72), which is a measure of impulsivity as a personality trait. Control of all of these variables is important, as drinkers with depression, anxiety, as well as high impulsivity symptoms have been shown to be at increased risk of developing alcohol dependence (73–75). Therefore, it can be seen that the participants of both groups did not exhibit any difference in terms of these variables (see **Table 1**). Indeed, based on these criteria, 60 undergraduate students were selected for the ERP study and classified as light (*n* = 30) or heavy (hazardous) drinkers (*n* = 30). Among these, 11 participants exhibiting EEG artifact contamination were removed. Therefore, the final groups were represented by 24 light and 25 hazardous drinkers. We obtained informed written consent from the participants after they were fully informed about the study. The local ethics committee of the Brugmann Hospital approved the study (“Comité d’Ethique Hospitalier CE 2010/156”). The participants were instructed to abstain from consuming alcohol in the 24 h before the ERP recording.

### Working Memory *n*-Back Task

**Figure 1:**
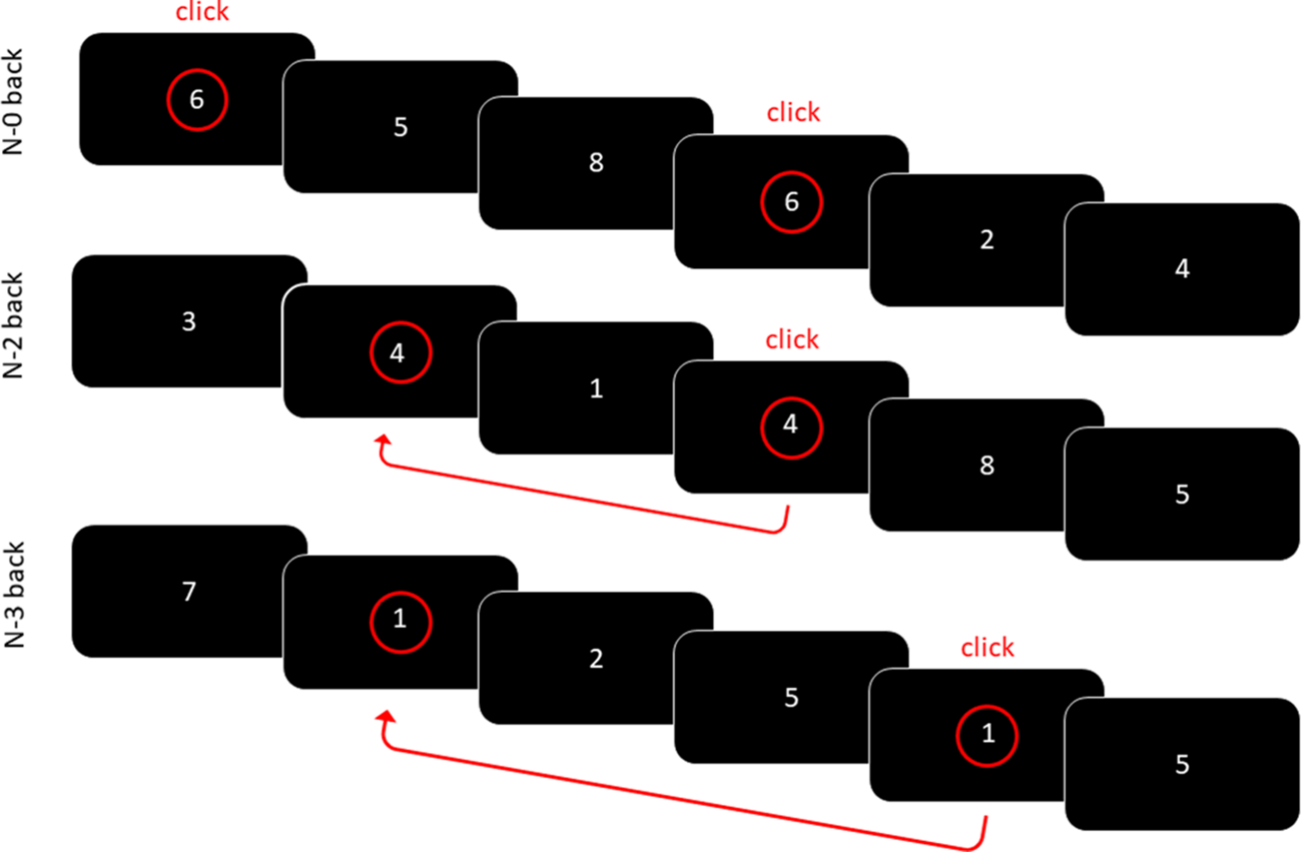
Visual N-back working memory task. In the N0 condition, the participants had to as quickly as possible detect the number 6. In the N2/N3 conditions, the participants had to press the button when the displayed number was identical to the number displayed two/three trials earlier.

WM performance and the underlying neural activity were measured using a visual *n*-back task under three different conditions. The stimuli were white numbers (Arial font, size 74) displayed on a black background on the center of the screen, presented successively in a pseudo-random order. In the vigilant/control zero-back (N0) condition, the subjects were asked to press a button with their right hand whenever the number “2” was displayed (block 1) or “6” (block 2). In the WM two-back (N2) and three-back (N3) conditions, the subjects had to press the button when the displayed number was identical to the number displayed two or three trials earlier (see **Figure 1** for an illustration). The subjects were successively administered two blocks in the N0 condition, then two blocks in the N2 and two blocks in the N3 conditions. This order was kept constant across the participants in order to ensure that all of the groups were exposed to exactly the same manipulation of tasks with increasing complexity (from N0 to N2 and then N3). Each N0 block consisted of a sequence of 80 trials (including 20 targets), while the N2 and N3 conditions consisted of a sequence of 86 (104) trials, respectively, also including 20 targets each. Each stimulus was displayed for 1,750 ms with an interstimulus interval of 250 ms. This way, 40 targets were available for each condition across the participants. The pseudo-random order ascertained that, in N0, two targets were not successively presented; and, in N2 and N3, that the same number was not repeatedly used as a target (but instead varied randomly from 1 to 9). All of the participants performed one practice block for each condition (N0, N2, and N3).

### EEG Recordings

During the ERP recordings, each participant sat alone in a darkened room, on a chair placed 1 m from the screen. EEG activity was recorded with 32 electrodes mounted on a Quick-Cap and placed in standard (based on the 10–20 system) and intermediate positions (Fpz, Fp1, Fp2, Fz, F3, F7, F4, F8, FC1, FC5, FC2, FC6, Cz, C3, C4, T7, CP5, CP1, CP2, CP6, T8, P7, P3, Pz, P4, P8, POz, O1, Oz, and O2). Recordings were made with a linked mastoid physical reference. The EEG was amplified with battery-operated ANT^®^ amplifiers with a gain of 30,000 and a bandpass of 0.01–100 Hz. The ground electrode (AFz) was positioned between Fpz and Fz along the midline. The impedance of all of the electrodes was maintained below 10 kΩ during all the experiments. EEG was recorded continuously at a sampling rate of 500 Hz with ANT Eeprobe software. Approximately 20% of the trials were contaminated (a cutoff of 30 mV was used to define trials that were contaminated either by eye movements or muscular artifacts), and they were eliminated offline in order to only analyze the artifact-free trials. Epochs starting 200 ms before the onset of the stimulus and lasting for 800 ms were created. The data were filtered with a 30-Hz low-pass filter. A baseline correction (from −200 to 0 ms) was computed. Only trials that were correctly performed were included in these averages [i.e., correct hits for targets, while hits for nontargets (false alarms) were eliminated]. Two parameters were coded for each stimulus: i) the condition (N0; N2; N3) and ii) the type of response (key press for targets, no key press for the other stimuli). This coding allowed us to compute different averages of ERP target stimuli. The averages were computed for each subject individually. Grand-averages were then computed for the three conditions (N0, N2, and N3) for each group (light vs. hazardous drinkers).

### Statistical Analyses

For the behavioral data, three ANOVAs were computed on the correct hits, the reaction times, and the false alarms with level (N0, N2, and N3) as within-subject variables, and group (light vs. hazardous drinkers) as a between-subject variable. The Greenhouse–Geisser correction was applied to all of the ANOVAs when necessary. For the ERP data, we first analyzed the two classical ERP components associated with the control N0 condition: 1) the P100 component, measured as a mean amplitude value over O1, Oz, and O2 electrodes in the latency range [80–140 ms] (55); and 2) the P300 component, measured as a mean amplitude value over P3, Pz, and P4 electrodes in the latency range [280–450 ms] (55). Then, as no group difference emerged on this baseline condition, the main analyses of this study consisted of subtracting it from the WM conditions (N2, N3) in order to isolate specific WM processes such as storage and manipulation (updating) (34, 42, 55). Subtractions “N2 minus N0” as well as “N3 minus N0” were then computed for each participant of each group and were subsequently grand-averaged. Significant effects were calculated at four selected electrode clusters [i.e., Frontal (mean of electrodes F3, F4, and Fz), Central (mean of Cz, C3, and C4), Parietal (mean of P3, Pz, and P4), and Occipital (mean of O1, Oz, and O2)] through Student’s *t*-tests (amplitude of the difference wave compared to zero from 0 to 800 ms) (76, 77). These *t*-values were significant at the level *p* < .01 if they were above 2.79/below −2.79 for the hazardous drinkers (significance threshold computed on the basis of a sample size of *n* = 25) or above 2.81/below −2.81 for the light drinkers [*n* = 24; see the critical values (percentiles) for the *t* distribution at https://faculty.washington.edu/heagerty/Books/Biostatistics/TABLES/t-Tables/]. Only spatiotemporal patterns whose *t*-values were significant *for at least 20 ms* were considered as relevant (76–78). All of the analyses were conducted with SPSS 20 software.

## Results

### Behavioral Data

The light and the hazardous drinkers were equivalent in terms of age, gender, depression (BDI-II scores), anxiety (STAI-trait and STAI-state scores), and impulsivity (UPPS total score; all *p*’s > 0.05). The two groups differed solely on “alcohol” variables, i.e., on the AUDIT-C subscore [*t*(47) = −10.836; *p* < 0.001], and on the DOW [*t*(47) = −6.236; *p* < 0.001], ADO [*t*(47) = −3.977; *p* < 0.001], as well as ADW [*t*(47) = −5.255; *p* < 0.001] variables. The complete demographic characteristics of the cohort are reported in **Table 1**. The ANOVAs revealed a significant principal effect of level on correct hits [F(2,94) = 197.549; *p* < 0.001; observed power = 1], reaction times [F(2,94) = 171.15; *p* < 0.001; observed power = 1], and false alarms [F(2,94) = 89.012; *p* < 0.001; observed power = 1]. This suggests a “complexity effect,” as the more difficult the task (N3 > N2 > N0), the more the participants made errors (fewer correct hits and more false alarms) and had longer response latencies. However, no significant effects of group or significant level × group interactions were found (all *p*’s > 0.05), suggesting that both groups performed the task similarly. Detailed analysis results are presented in **Table 2**.

**Table 2:** The ANOVAs revealed a significant principal effect of level on correct hits [F(2,94) = 197.549; *p* < 0.001; observed power = 1], reaction times [F(2,94) = 171.15; *p* < 0.001; observed power = 1], and false alarms [F(2,94) = 89.012; *p* < 0.001; observed power = 1]. No significant effects of groups or significant level × group interactions were found (all *p*’s > 0.05), suggesting that both groups performed the task similarly.

	Level	Light drinkers	Hazardous drinkers
Correct hits (/40)	N0	40 ± 0	39.88 ± 0.3
	N2	33.79 ± 2.6	35.2 ± 2.08
	N3	28.25 ± 5.2	28.04 ± 4.8
Reaction times	N0	422 ± 54.4	423 ± 71.4
	N2	586 ± 110.2	549 ± 98.6
	N3	741 ± 148.3	777 ± 141.4
False alarm	N0	0.13 ± 0.3	0.2 ± 0.5
	N2	3.12 ± 2.3	2.96 ± 1.5
	N3	7.79 ± 4.5	8.28 ± 5.5

### Event-Related Potential Data

**Figure 2:**
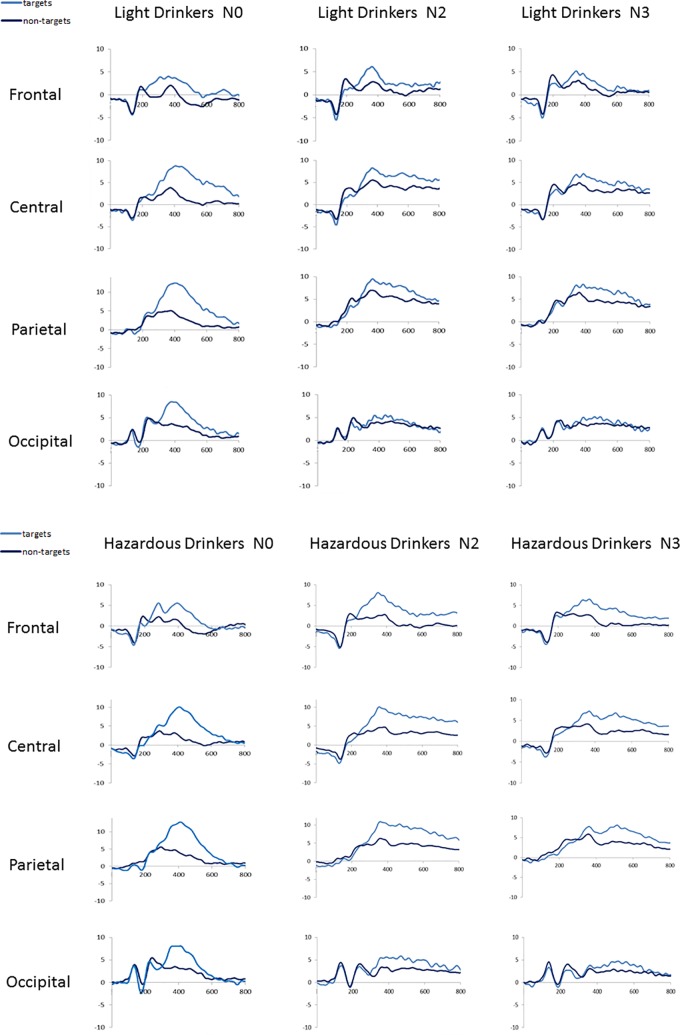
Waveforms recorded at frontal (mean amplitudes for F3, Fz, F4), central (C3, Cz, C4), parietal (P3, Pz, P4), and occipital (O1, Oz, O2) sites for the light (*n* = 24) and the hazardous (*n* = 25) drinkers on each condition (N0, N2, N3) for target and nontarget trials.

At a technical level, we first ensured that the same number of trials was included in ERP analyses for both groups across conditions. An ANOVA 2 × 3 with group (light vs. hazardous drinkers) as a between-subject variable and condition (N0, N2, N3) as a within-subject variable was computed. As only correct hits for targets were entered in ERP analyses, we were able to show a main condition effect [F(2,94) = 60.582; *p* < 0.001; observed power = 1], indexing an increased number of errors as a function of task complexity [mean number of trials ± SD: N0 Light: 29 (7.6), Hazardous: 32 (6.8); N2: Light: 22 (6.9), Hazardous: 25 (7.9); N3: Light: 18 (7), and Hazardous: 20 (7)]. However, this complexity effect was not modulated by the group [no group effect: F(1,47) = 2.575; *p* = 0.115; no interaction condition × group: F(2,94) = 0.231; *p* = 0.779], suggesting that a similar signal-to-noise ratio was ensured for each condition between groups. Waveforms recorded on target and nontarget trials in each condition (N0, N2, and N3) are shown in **Figure 2**. As expected, the targets involved widespread higher amplitudes than the nontargets [e.g., Ref. (55)].

We then compared P100 and P300 amplitudes on the baseline N0 condition between the light and the hazardous drinkers. We used two ANOVAs with group (light vs. hazardous drinkers) as a between-subject variable. No significant difference emerged (all *p*’s > 0.05). Therefore, as expected [e.g., Ref. (42)], we were able to compute “N2 minus N0” as well as “N3 minus N0” subtractions.

**Figure 3:**
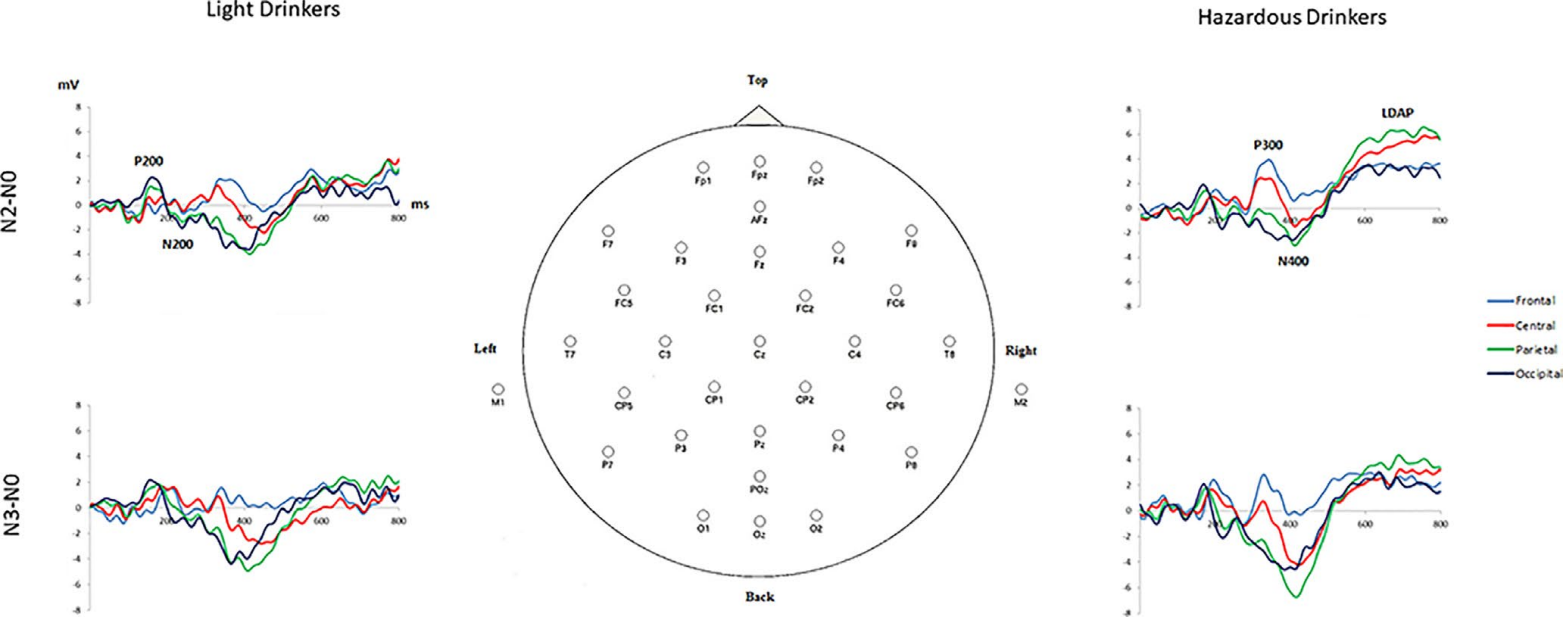
Subtracted grand-average waveforms “N2 minus N0” and “N3 minus N0” at frontal (mean amplitudes for F3, Fz, F4), central (C3, Cz, C4), parietal (P3, Pz, P4), and occipital (O1, Oz, O2) sites for the light (*n* = 24) and the hazardous (*n* = 25) drinkers.

The subtraction “N2 minus N0” revealed three main components in both groups: 1) a widespread positivity (with maximal amplitudes visible at occipital sites) associated with a negativity maximally recorded at occipital sites around 150–250 ms: such a pattern exhibited high similarity with the P200/N200 recorded by Missonnier and colleagues (55); 2) a positive activity around 280–400 ms, mainly visible at frontal sites, that can refer to the well-known P300 component, as in Johnson and colleagues’ (79) study; and 3) a large negativity around 300–500 ms associated with a long-lasting positivity starting around 500 ms on all of the electrodes (Fz, Cz, Pz, and Oz) that can be linked to the “old/new” N400/P600 memory effect (80). In the same way, the subtraction “N3 minus N0” also revealed these three main components. This is illustrated in **Figure 3** and **Table 3**.

**Table 3:** Mean amplitude values (± SD) for the main ERP components resulting from “N2 minus N0” and “N3 minus N0” subtractions on time intervals and on sites of maximally recorded amplitudes for the light and the hazardous drinkers.

			Light (N2−N0)	Hazardous (N2−N0)
**P200**	100–200 ms	Occipital	1.15 (± 0.82)	0.77 (± 0.68)
**N200**	200–300 ms	Occipital	−1.16 (± 0.35)	−0.96 (± 0.47)
**P300**	300–400 ms	Frontal	1.57 (± 0.62)	2.80 (± 0.90)
**N400**	300–500 ms	Parietal	−2.45 (± 0.97)	−1.19 (± 0.96)
**LDAP**	500–800 ms	Parietal	1.89 (± 1.03)	5.14 (± 1.52)
			Light (N3−N0)	Hazardous (N3−N0)
**P200**	100–200 ms	Occipital	1.16 (± 0.74)	0.40 (± 0.96)
**N200**	200–300 ms	Occipital	−1.03 (± 0.30)	−1.57 (± 0.60)
**P300**	300–400 ms	Frontal	0.76 (± 0.49)	1.36 (± 0.85)
**N400**	300–500 ms	Parietal	−3.34 (± 1.14)	−4.17 (± 1.62)
**LDAP**	500–800 ms	Parietal	1.25 (± 1.08)	2.98 (± 1.22)
LDAP, Late Directing Attention Positivity.				

In order to compare these “subtracted waveforms” (N2 minus N0; N3 minus N0), between groups, we submitted these data to Student’s *t*-tests (amplitude of the difference wave compared to zero from 0 to 800 ms) (76, 77) in order to isolate specific spatiotemporal electrophysiological patterns devoted to the WM processes involved in our task (such as storage and updating) (34, 42, 55). To achieve this, and to deal with the multiple comparisons that we computed, we considered that patterns for which the *t*-values were above 2.79/below −2.79 (*p* < .01) for the hazardous drinkers (*n* = 24) or above 2.81/below −2.81 (*p* < .01) for the light drinkers (*n* = 25) were significant *only if they lasted for at least 20 ms* (76–78).

**Figure 4:**
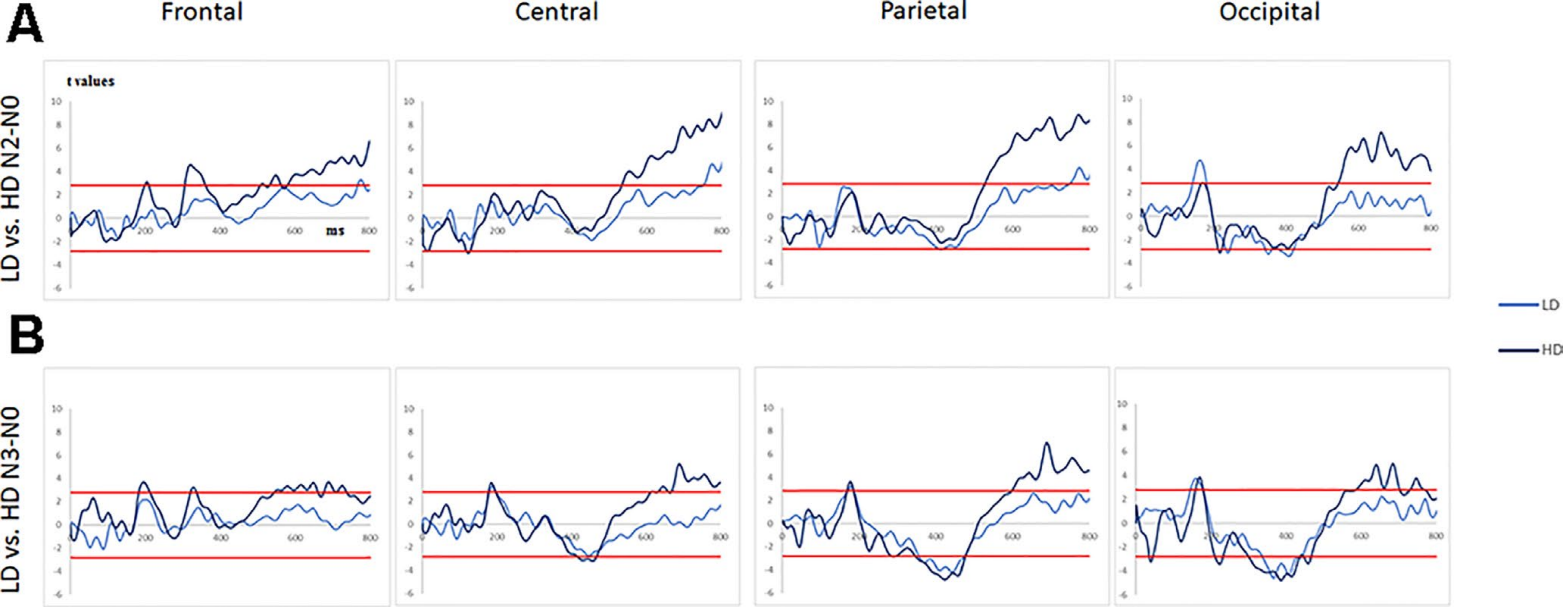
**(A)** T-values obtained for the subtraction “N2 minus N0” (significance levels are represented by red lines) for the light and the hazardous drinkers at frontal, central, parietal, and occipital sites. **(B)** *T*-values obtained for the subtraction “N3 minus N0” (significance levels are represented as red lines) for the light and the hazardous drinkers at frontal, central, parietal, and occipital sites.

For “N2 minus N0,” the light drinkers exhibited 1) at frontal sites, no significant difference while the hazardous drinkers exhibited three patterns of significant “difference” activities at ms [306–361], [533–564], and [581–819]; 2) at central sites, a small late difference at ms [751–819], while this difference was more sustained in the hazardous drinkers at ms [532–819]; 3) at parietal sites, a similar pattern to the one described at central sites, i.e., a significant activity around [747–819] ms for the light drinkers and a more sustained one in the hazardous participants around [527–819] ms; and 4) at occipital sites, two significant differences, at [143–180] ms and [379–421] ms intervals, that were not observable in the hazardous drinkers, who always exhibited a sustained later activity around ms [545–819]. The results are shown in **Figure 4A** and **Table 4A**.

**Table 4A:** Statistically significant time intervals (in ms) for the subtracted waveforms exhibited at frontal, central, parietal, and occipital sites for the subtraction “N2 minus N0.” A significant interval was considered as relevant (in green) when it lasted for at least 20 ms (76–78). Other intervals (in red) were neither considered nor discussed. P for positive activity; N for negative activity.

	Light drinkers (*n* = 24)	Hazardous drinkers (*n* = 25)
Frontal	P [769; 786] P [806; 819]	P [198; 209] P [306; 361] P [508; 520] P [533; 564] P [581; 819]
Central	P [751; 819]	P [11; 15] P [117; 127] P [532; 819]
Parietal	P [406; 419] P [747; 819]	P [527; 819]
Occipital	P [143; 180] N [234; 245] N [343; 361] P [379; 421]	P [167; 174] P [213; 223] P [545; 819]

For “N3 minus N0,” one can observe 1) at frontal sites, no significant difference for the light drinkers while the hazardous drinkers exhibited three patterns of significant “difference” activities at ms [182–211], [544–659], and [676–736]; 2) at central sites, no significant difference for the light drinkers while the hazardous drinkers exhibited three significant intervals at ms [415–467], [632–659], and [668–819]; 3) at parietal sites, a significant activity around [356–475] ms for the light drinkers and two for the hazardous participants around [346–476] and [597–819] ms; and 4) at occipital sites, two significant differences, at [146–178] and [337–443] ms intervals, that emerged for the light drinkers while the hazardous drinkers exhibited four significant patterns of activities at ms [211–231], [300–435], [585–702], and [730–769]. The results are illustrated in **Figure 4B** and **Table 4B**.

**Table 4B:** Statistically significant time intervals (in ms) for the subtracted waveforms exhibited at frontal, central, parietal, and occipital sites for the subtraction “N3 minus N0.” A significant interval was considered as relevant (in green) when it lasted for at least 20 ms (76–78). Other intervals (in red) were neither considered nor discussed. P for positive activity; N for negative activity.

	Light drinkers (*n* = 24)	Hazardous drinkers (*n* = 25)
Frontal	Ø	P [182; 211] P [320; 335] P [544; 659] P [676; 736]
Central	P [180; 196]	P [177; 196] N [415; 467] P [613; 622] P [632; 659] P [668; 819]
Parietal	P [169; 186] N [356; 475] P [808; 819]	P [168; 187] N [283; 295] P [346; 476] P [597; 819]
Occipital	P [146; 178] N [337; 443]	N [36; 45] P [159; 178] N [211; 231] N [300; 435] N [447; 466] P [585; 702] P [730; 769]

Overall, the hazardous drinkers exhibited enhanced amplitude activities compared to the light drinkers, to perform N2 and N3-back conditions. More precisely, the hazardous drinkers exhibited higher amplitude differences, mainly at frontal P300 and widespread P600 components, whereas the light drinkers exhibited enhanced amplitudes around the P200 and N400 components. It should also be noted that, even though the hazardous drinkers exhibited a higher number of significant activities in the N3-back condition compared to the N2-back condition (suggesting incremental activity with task complexity), group differences between the light and the hazardous drinkers were of higher amplitudes for the N2 minus N0 condition than for the N3 minus N0 one. This suggests that the hazardous drinkers exhibited higher processing intensity throughout the information-processing stream, notably around the P300 and the late directing attention positivity (LDAP) components, while the light drinkers can just increase early visual attention (P200) in order to obtain a better memory trace (N400) to deal with the *n*-back task implying different cognitive loads.

## Discussion

Although many *n*-back studies have not reported any significant difference between healthy participants and excessive alcohol drinkers, significant neural differences have been found indexing compensatory neural processing during variation in the cognitive load (40–44, 46). Moreover, these neural differences appear to be observable throughout the information processing stream when electrophysiological measures (characterized by a better temporal resolution) are used (52–56). In the present ERP study, and for the first time to our knowledge, increasing memory load (N2 and N3-back tasks) has been placed on light and hazardous drinkers.

The main result of the present study is that, even though the performances were equal between the groups, the hazardous drinkers exhibited more intense and widespread activities than the light drinkers. These data are in total agreement with previous data obtained in our lab through an fMRI study (42), in which hazardous drinkers exhibited higher bilateral activity in the pre-supplementary motor area as well as specific positive correlations between the number of alcohol doses consumed per occasion and higher activity in the dorsomedial PFC, and between the number of drinking occasions per week and higher activity in cerebellum, thalamus, and insula while performing the N2 memory task. The present study extended these results, as it showed that 1) these enhanced activities are also present in the N3-back task; and 2) as a result of the optimal resolution of ERPs, it specified the *temporal dynamic* of these increased activities.

At the behavioral level, our results confirmed that the N3 condition was considerably more difficult than the N2-back task, but also that no difference was observable between the groups (as expected) (42). At the ERP level, as the baseline N0 condition was similar across the groups, we computed subtraction “N2 minus N0” and “N3 minus N0” to isolate WM processes (34). By visual inspection, three patterns of activities could be discerned: one around 150 to 250 ms with a maximal activity at posterior sites (P200/N200 complex), one around 300 to 400 ms at frontal sites (P300), and one around 400 to 800 ms as a late positive potential (N400/P600 complex). Therefore, Student’s *t*-tests (amplitude of the difference wave compared to zero from 0 to 800 ms) (76, 77) were applied in order to assess statistically significant differences among these three spatiotemporal patterns of activities between the light and the hazardous drinkers.

According to a “functional compensation view,” increases in activation reflect “attempted” or “successful” compensation for these deficits during more complex cognitive tasks (47). These changes in cerebral responses may be considered, at the preventive level (particularly for young drinkers), as vulnerability factors for the development of adult SUD (42), but also stressed the importance, at a more clinical level, to consider such WM processes (such as the ability to deal with a high cognitive load) in the management of alcohol dependence. Some studies aiming to train WM efficiency in excessive alcohol users have already been published, disclosing encouraging results (27, 28). Moreover, it has also been shown that prior WM training with a high memory load interferes with the reconsolidation of alcohol-related memories in a sample of nontreatment-seeking heavy drinkers (81). However, more studies tagged dual-process mechanisms [cue reactivity/inhibition; for instance, Refs. (17, 18)]. As WM capacity has been shown to impact cognitive control of impulsivity by way of keeping future goals in mind when making decisions when faced with rewarding/arousing distractions (19), a point that perfectly fits with the dual-process model of cognitive control, further studies aiming to develop cognitive training procedure for alcohol-dependent patients should include the WM process.

Also, it is worth noting that the ERP data we obtained are in line with several previous ERP studies. First, the P200/N200 component has already been described by Missonnier and colleagues (55), by subtracting ERP waveforms from memory-free control tasks (detection) from memory tasks (1-back and 2-back tasks), its amplitude increasing significantly in healthy subjects with higher memory load (2-back vs. 1-back). At the functional level, this complex was interpreted as an *intermediate* phase, as short-term storage should directly follow pure sensory-driven processes (such as the P100) and precede execution-related processes (300 ms or later). Therefore, the P200/N200 complex could refer to the visual encoding of the stimulus, translated into its corresponding phonological representations (1), which is created and stored in the posterior parietal cortex, remains active for a few seconds, and constitutes *the storage function of verbal working memory* (82). It needs to be emphasized that the light drinkers exhibited higher responses (for the P200 in the N2-back task) than the hazardous ones. Usually, when task-relevant images are displayed, the early/sustained attention increases, thereby increasing the impact of the stimuli [e.g., Ref. (83)]. This could suggest that the light drinkers generally exhibited an *enhanced early visual attentional process* to ease task performance compared to the hazardous drinkers (consistent with a recollection process aided by visual imagery) (36). Secondly, similarities were also found with a study by Johnson et al. (79), which focused on the *refreshing* process. Refreshing is thought to be a key process for selecting, maintaining, and manipulating information within WM (84), and is, therefore, a critical component in tasks that require manipulation such as updating (e.g., *n*-back) (85). In that study, ERP analyses showed that a typical refresh task does have a distinct electrophysiological response compared to a control condition, and it includes at least two main temporal components: an earlier (∼400 ms) positive peak reminiscent of a P3a/P3b response and a later (∼800–1,400 ms) sustained positivity over several sites reminiscent of the late directing attention positivity (P600 or LDAP) (79). In our study, we found a positive component around 280 to 400 ms, and one around 500 to 800 ms as a late positive potential. These two distinct component cognitive processes are consistent with a two-phase model predicted from fMRI: the first phase referring to the initiation of an appropriate nonautomatic cognitive or motor action based on the interpretation of a cue, and the second reflecting top-down modulation carrying meaningful information about currently active mental representation (79). In this view, it seems reasonable to draw some connections between these two components and the P3 family of responses (typically occurring around 280–500 ms) (86) and the P600 or LDAP (typically arising around 500 ms post-cue) (87). On the one hand, our component around 280 to 400 ms could be related to both the P3a, which is related to the initial orientation to and evaluation of a stimulus, driven primarily by prefrontal regions (88), and the P3b, which appears to be related more to the resolution of uncertainty about stimuli and the concomitant updating of expectancies or context, potentially engaging additional attentional or memory processes, and driven primarily by temporoparietal activity (86, 88). On the other hand, our late positive component from 500 to 800 ms may be seen as similar to a P600 or LDAP, a late positive potential associated with perceptual attention, lasting up to several hundred milliseconds. It has been interpreted as arising from the anticipatory top-down modulation of visual regions in response to the refreshing of a visual representation [e.g., Ref. (89)]. Such WM processes required more intense and sustained activities in the hazardous drinkers compared to the light ones, therefore suggesting a type of vulnerability of these cognitive processes. Thirdly, it is also worth noting that such an LDAP has also been previously linked to an N400 component. Indeed, Finnigan and colleagues (80) recorded ERPs while subjects made old/new recognition judgments on new unstudied words and old words that had been presented in the study either once (“weak”) or three times (“strong”). They showed that the N400 component was found to be modulated in a graded manner by the memory trace strength (i.e., an “N400 strength effect”) while the amplitude of the LDAP was sensitive to confidence in the decision accuracy. In the present study, the light drinkers exhibited higher amplitudes for this N400 component compared to the hazardous drinkers, suggesting a more intense memory trace.

Overall, one of the main strengths of ERPs is to be able to provide *a dynamic temporal view* of a cognitive process. Using visual *n*-back WM with different cognitive loads (N2-back, N3-back) appears to reveal such an information-processing stream, impacted by alcohol consumption: aside from physical processing of visual stimuli, participants have 1) to translate, encode, and store visual stimuli in short-term verbal memory (i.e., the P200/N200 complex); 2) to orient attention to stimuli (P3a), update short-term memory, and make decisions (P3b); and 3) this decision being impacted by the memory trace strength (N400) and confidence in the decision accuracy (LDAP). *To perform the task at the same level as the light drinkers, the hazardous drinkers exhibited a higher processing intensity throughout the information-processing stream, notably around the P300 and the LDAP components, while the light drinkers could merely increase early visual attention (P200) in order to obtain a better memory trace (N400).* This increment in the neural resources needed to accomplish a more and more complex task can be seen as a compensation strategy. According to a “functional compensation view,” concomitant increases in activation reflect “successful” compensation for these deficits (47). Indeed, due to neuronal loss induced by the neurotoxic effect of alcohol, excessive drinkers need more resources to successfully perform a task. This could imply that 1) once the threshold of available resources is reached (for instance, by making the task more and more complex), a behavioral deficit will appear; and 2) with a less efficient WM load process, excessive drinkers may have fewer resources to plan long-term goals (e.g., be healthy), increasing propensity (i.e., decreasing cognitive control) towards an immediate reward (e.g., a drink). Therefore, it is important to highlight such data for at least two main reasons. First, at a preventive level, it seems important to stress that, *at a stage at which behavioral manifestations are not yet observable,* social heavy drinking is not just trivial social fun, as it induces substantial neural modifications subtending cognitive functions such as WM processes that may impact continuation of excessive alcohol consumption (for instance, by minimizing the impact of long-term consequences). And second, at a clinical level, training WM load capacity may reduce future alcohol consumption by increasing attention toward long-term goals, by increasing control toward immediate rewards that are not relevant to long-term prospects, and by facilitating reconsolidation of alcohol-related memories [e.g., Refs. (27, 28, Kaag et al., 2017)].

Clearly, we are fully aware that our present findings do not allow us to map ERP phenomena directly onto specific cortical areas, and that the relationships that we present above (even though theoretically grounded) are speculative. Such clear associations can, for instance, be obtained through combined ERP-fMRI studies [e.g., Ref. (90)]. We are also aware that it is not possible, from the present study, to completely discount the possibility that the differential effects observed for the hazardous drinkers are pre-morbid in nature, i.e., they existed prior to any alcohol consumption. In this view, further longitudinal studies should be designed in order to verify whether the emergence of brain differences in heavy drinkers did or did not follow the onset of drinking habits. Also, even though the N3-back tasks were more difficult than the N2-back tasks at the behavioral level, electrophysiological group differences between N2 and N0 conditions revealed *higher amplitude differences* than those between N3 and N0 conditions. This could be due to an “order effect,” as the participants were always exposed to N2-back tasks *before* N3 ones. This ensured that all of the participants were exposed to conditions that were entirely similar. However, the participants could also develop a strategy to perform the N2-back condition and then apply it in the N3-back tasks so that the latter could require fewer neural resources than if they had been performed first (i.e., when the participants were still “naive” and have to adapt to the task). A fatigability effect cannot be excluded either. Therefore, further studies should alter the order of the presentation of these different conditions in order to be able to directly compare N3-back and N2-back tasks. Indeed, such a comparison would be biased in the present study as neural activities recorded in the N3-back condition appear to be decreased compared to the N2-back ones due to a type of “habituation” effect. This way, one could investigate whether differences between light and hazardous drinkers increase as a function of the cognitive load.

## Ethics Statement

The local ethics committee of the Brugmann Hospital approved the study (“Comité d’Ethique Hospitalier CE 2010/156”).

## Author Contributions

ES and CD contributed equally to the paper (coauthors). They acquired and processed the data and wrote the paper. XN and CK participated to writing the paper. SC participated to task design and paper writing.

## Funding

The authors were funded by the Belgian Fund for Scientific Research (F.N.R.S., Belgium) and the Brugmann Foundation (CHU Brugmann, Brussels, Belgium), although these funds did not exert any editorial direction or censorship on any part of this article.

## Conflict of Interest Statement

The authors declare that the research was conducted in the absence of any commercial or financial relationships that could be construed as potential conflicts of interest.
